# Effect of lixisenatide on arterial stiffness in people with type 2 diabetes and kidney disease: Results of a randomised controlled trial

**DOI:** 10.1111/dom.70481

**Published:** 2026-02-18

**Authors:** Nikolaos Fountoulakis, Panagiotis Pavlou, Dimitra Stathi, Aicha Goubar, Antonella Corcillo, Maria Flaquer, Salma Ayis, Luigi Gnudi, Janaka Karalliedde

**Affiliations:** ^1^ School of Cardiovascular and Metabolic Medicine & Sciences King's College London London UK; ^2^ School of Population Health & Environmental Sciences King's College London London UK

**Keywords:** aortic pulse wave velocity, arterial stiffness, diabetic kidney disease, GLP‐1 receptor agonist

## Abstract

**Objective:**

People with chronic kidney disease (CKD) and diabetes are at high risk of cardiovascular disease (CVD). Aortic pulse wave velocity (Ao‐PWV) is an independent predictor of CVD. Cardiovascular outcome trials (CVOTs) with glucagon like peptide‐1 receptor agonist (GLP‐1 RA) class demonstrate notable differences, with lixisenatide having neutral effects as compared to longer acting GLP‐1 RA. It is unknown if shorter acting GLP‐1 RA have an impact on Ao‐PWV and if this may explain the discordance observed in GLP‐1RA CVOTs.

**Materials and Methods:**

We studied people with type 2 diabetes and CKD in a proof‐of‐concept single centre, randomised, double‐blind parallel‐group placebo‐controlled study that evaluated 24 weeks' treatment with lixisenatide as compared to placebo on the primary endpoint of Ao‐PWV.

**Results:**

In total, 101 participants (male 66%) were randomised of whom 90 were eligible for analyses (lixisenatide [*n* = 47] and placebo [*n* = 43]). Ao‐PWV did not change significantly from baseline after 24 weeks of treatment with final mean (95% confidence intervals) of 9.65 (9.17, 10.13) m/s with lixisenatide and 9.96 (9.45, 10.46) m/s with placebo, *p* = 0.38. Similarly, no significant changes were observed in cardio‐renal risk biomarkers including albuminuria and Klotho levels. HbA1c decreased with lixisenatide as compared to placebo.

**Conclusions:**

In people with CKD and type 2 diabetes the use of short‐acting GLP‐1 RA lixisenatide did not significantly influence Ao‐PWV. Further studies are needed to understand mechanisms that may explain discordance in CVOTs results observed with GLP‐1 RA.

**Clinical Trial Registration:**

ISRCTN: ISRCTN97699312; EudraCT/CTIS number: 2016‐001758‐17.

## 1 BACKGROUND

Glucagon‐like peptide‐1 receptor agonists (GLP‐1 RAs) have demonstrated cardiovascular disease (CVD) benefits in large randomised controlled trials in people with type 2 diabetes (T2D) [1, 2]. However there are some notable differences within the class with very short acting agents such as lixisenatide and exenatide, not demonstrating positive benefits or having mixed results in cardiovascular outcome trials (CVOTs) as compared to longer acting daily (liraglutide) or weekly agents (such as semaglutide and dulaglutide) [3, 4, 5, 6, 7, 8, 9]. Even accounting for differences in CVOT designs and inclusion criteria where broadly similar participants were included, notable differences between longer acting agents such as weekly albiglutide, which demonstrated CVD benefits are observed as compared to shorter acting agents such as lixisenatide which had no CVD benefits observed in a CVOT [10, 11]. People with chronic kidney disease (CKD) and diabetes are at enhanced CVD risk [12] and a recent study in people with T2D and CKD demonstrated CVD and renal benefits with the longer acting GLP‐1 RA semaglutide [13].

Arterial stiffness as measured by aortic pulse wave velocity (Ao‐PWV) is an independent predictor of CVD and renal outcomes in people with T2D [14, 15, 16]. In a meta‐analysis including more than 17,000 people pooled from multiple studies, addition of Ao‐PWV to risk models enabled better and precise identification of high‐risk populations that could benefit from more enhanced CVD risk factor management [17].

Reversibility of Ao‐PWV is an important modifiable risk factor for reducing the burden of CVD and mortality in people with CKD, while increased Ao‐PWV is also independently associated with deterioration in renal function [16, 18]. A large meta‐analysis demonstrated that an increase in Ao‐PWV by 1 m/s corresponded to an age‐, sex‐, and risk factor‐adjusted risk increase of 14%, 15%, and 15% in total CVD events, CVD mortality, and all‐cause mortality, respectively [19].

To further evaluate the role of shorter acting GLP‐1 RA on Ao‐PWV we performed a single centre, randomised, double‐blind parallel‐group placebo‐controlled study that evaluated the effect of 24 weeks' treatment with short acting GLP‐1 RA lixisenatide as compared to placebo on the primary endpoint of Ao‐PWV.

## 2 MATERIALS AND METHODS

This was a single centre, randomised, double‐blind parallel group placebo‐controlled study that evaluated if 24 weeks' treatment with lixisenatide as compared to placebo had an effect on the primary endpoint of Ao‐PWV. Lixisenatide or placebo treatment was added on to existing medical therapy for the management of T2D and CKD including renin angiotensin system (RAS) inhibition which was the main standard of care for CKD at the time of study design. Informed consent was obtained from all trial participants.

### 2.1 Inclusion criteria

Inclusion criteria included people with T2D age over 35 years with clinical diagnosis of CKD on a maximum tolerated and stable dose of ACE‐inhibitor or angiotensin receptor blocker in the preceding 3 months. All participants needed to have an estimated glomerular filtration rate (eGFR) more than 30 mL/min. People with a history of a CVD event within the past 12 months, non‐diabetic renal disease, recent or current use of GLP‐1 RA, history of connective tissue disease or inflammatory arthritis, uncontrolled hypertension (systolic blood pressure [SBP] and diastolic blood pressure (DBP) greater than 180 and 100 mmHg, respectively), pregnancy and lactation were excluded.

Other exclusion criteria included history of pancreatitis, active gastrointestinal (GI) or biliary disease, planned major GI surgery that can/could affect upper GI function, history or family history of thyroid cancer or multiple endocrine neoplasia‐2, known allergy/intolerance to GLP‐1 RA, metacresol or any of the study medication or placebo components. Sodium‐glucose co‐transporter 2 (SGLT‐2) inhibitors were permitted; however, at the time of study design, this class of agent was not standard of care for kidney disease. Finerenone, a non‐steroidal mineralocorticoid receptor agonist (nsMRA) with a current indication for CKD in diabetes, was not available for clinical use when this study was conducted.

The trial was conducted in compliance with the principles of the Declaration of Helsinki (1996), and in accordance with all applicable regulatory requirements. Clinical trial registry number/EudraCT Number: 2016‐001758‐17. The study was approved by Guy's Research Ethics Committee and the Medicines and Healthcare Products Regulatory Agency UK. All participants provided informed consent and were recruited from the outpatient diabetes clinics. The study was funded by a research grant from Sanofi‐Aventis.

### 2.2 Interventions

Following a screening visit, participants were randomised by means of a computer‐generated random sequence to either lixisenatide 10 mcg once daily increased to 20 mcg after 2 weeks or placebo. The primary endpoint of the trial was change in Ao‐PWV from baseline to week 24.

It was estimated that 60 participants per treatment group would provide 90% power at the 5% level (two‐sided) to detect a difference in Ao‐PWV between lixisenatide and placebo of 1.0 m/s with a population standard deviation of 1.5 m/s, which was assuming a 20% dropout rate during the study. The number without this estimated dropout that was needed to observe the effect size of 1.0 m/s was 52 participants per treatment group (104 in total) [20]. We have previously demonstrated a blood pressure independent 1 m/s reduction in Ao‐PWV is detectable following 24 weeks' treatment with Valsartan in participants with T2D CKD [20]. Recent data has demonstrated that other GLP‐1 RA treatments are associated with a reduction in Ao‐PWV of between 0.5 and 1.9 m/s, which was observed in sample sizes that were between 20 and 56 participants who were treated for 24 weeks or less [21, 22].

Secondary endpoints included changes in albuminuria, central aortic blood pressure, and biomarker of arterial ageing sKlotho, which has been associated with Ao‐PWV and CKD progression in T2DM [23]. Other secondary endpoints included changes in haemoglobin A1c (HbA1c), changes in serum electrolytes (sodium, potassium), lipids (cholesterol, high‐density lipoprotein [HDL], low‐density lipoprotein [LDL], triglycerides), haematocrit and haemoglobin.

All measurements and procedures were performed by the investigator blinded to treatment allocation with the participant in the fasted state and having refrained from nicotine, alcohol, and caffeine for at least the previous 10 h. Brachial blood pressure was measured in triplicate in the supine position by an automated sphygmomanometer (Omron Digital Blood Pressure Monitor HEM 907, Bannockburn, IL).

Ao‐PWV was determined from carotid and femoral pressure waveforms obtained noninvasively by applanation tonometry (Millar tonometer, Millar Instruments, Houston, TX) using the Sphygmocor system (Atcor, Sydney, Australia) as previously described [23]. Participants were in the supine position, rested for 15 min, and measurements were taken by a single observer in a temperature‐controlled (22°C), quiet room. Waveforms were referenced to a concurrently recorded electrocardiogram, and carotid to femoral transit time (ΔT) was calculated from the foot‐to‐foot time difference between carotid and femoral waveforms. The distance between the surface markings of the sternal notch and femoral artery was used to estimate the path length between the carotid and femoral arteries (L), and Ao‐PWV was computed as L/ΔT. The within‐subject SD of Ao‐PWV assessed using this method in our laboratory is 0.5 m/s. The intraobserver coefficient of variation was 3.5%. The mean Ao‐PWV was calculated as the average of three measurements. Central blood pressure determinations including the aortic augmentation index (AIx) were also measured using the same methods.

Urine albumin concentration was measured by immunoturbidimetry using a CobasMiras Plus analyzer (Roche Diagnostics, Rotkreuz, Switzerland) from three‐timed overnight urine collections, and the median albumin excretion rate was calculated.

Serum total cholesterol (enzymatic colorimetry) and creatinine levels were also measured using a Cobas Mira Plus analyzer. Plasmas‐Klotho (Immuno‐Biological‐Laboratories, Hamburg, Germany) was measured in duplicate by enzyme‐linked immunoassay from samples stored at −80°C [24]. Blood samples were immediately centrifuged at 1500 g at 4°C for 10 min, and the supernatant fractions were stored at −80°C with no freeze–thaw cycles before analysis. HbA1c was measured by boronate affinity high‐performance liquid chromatography (CLC330; Primus, Kansas City, MO). eGFR was determined using the Modification of Diet in Renal Disease formula with values reported in ml/min/1.73 m^2^ [25]. Plasma sKlotho was measured by Immuno‐Biological‐Laboratories, Hamburg, Germany.

The trial was suspended during the COVID‐19 pandemic when the number of participants in each group was *N* = 51 for lixisenatide and *N* = 50 for placebo. There was no difference in the timing of baseline and follow‐up assessments between early and late recruits as no study visits or recruitment was performed during the pandemic. Furthermore, no follow‐up data are missing due to the COVID‐19 pandemic as all participants who remained in the study for its entirety completed their planned 24‐week follow‐up visits prior to the trial being suspended at the onset of the pandemic. A total of 90 people were eligible for the final modified intention‐to‐treat (mITT) analyses (lixisenatide *n* = 47 and placebo *n* = 43). The trial was not restarted post pandemic due to supply problems with the investigational product.

### 2.3 Statistical analysis

Descriptive statistics were used for the analysis of demographic and clinical features. Data were compared using an unpaired *t* test (for continuous normally distributed variables), Mann–Whitney test (for continuous variables not normally distributed) and χ^2^ test (for categorical variables). The change in Ao‐PWV was analysed using an analysis of covariance with baseline value as covariate. Endpoint was defined as the last available post‐randomisation measurement of endpoints in mITT analysis. Participants with any data following randomisation were included in the mITT analysis which was defined as receipt of at least one dose of study medication and availability of post‐randomisation Ao‐PWV measurements. Variables were tested for normality by Shapiro test and Q–Q plots and further mean, standard deviation and 95% confidence intervals were calculated for the normally distributed variables and median and interquartile range for the non‐normally distributed variables. Missing data were handled by last available post‐randomisation observation carried forward (LOCF). All statistical analysis was done within Rstudio (version 1.3.1073) under R version 4.0.2 (R Foundation for Statistical Computing, Vienna, Austria). A two‐tailed *p* value <0.05 was considered significant.

## 3 RESULTS

We initially assessed 143 participants for eligibility, of which 42 were excluded (Figure S1, Supporting Information). In total, 101 participants were randomised to lixisenatide (*n* = 51) or placebo (*n* = 50), of whom 90 were eligible for the final mITT analyses (lixisenatide *n* = 47 and placebo *n* = 43) (see Figure S1). Of this cohort, 64.4% were males and 35.6% females with T2D, mean (standard deviation) age of 61.8 (9.3) years, 46.7% of Black heritage and 45.6% of Caucasian, with the remainder from Asian, other and mixed heritages (Table 1). All participants were on RAS inhibition. Selected baseline characteristics and data are shown in Table 1. The primary reasons for dropout were participant choice and inability to adhere to the study visit schedule, with one participant withdrawing due to health reasons unrelated to the investigational medicinal product (IMP) (placebo arm). Of those who withdrew, *n* = 7 were in the lixisenatide arm and *n* = 10 in the placebo arm.

**TABLE 1:** Baseline characteristics and data for selected variables in people with T2D and CKD randomised to lixisenatide and placebo.

	Lixisenatide, *n* = 47	Placebo, *n* = 43	Total, *n* = 90
	*n* (%)	*n* (%)	*n* (%)
Age mean (SD)	61.0 (8.3)	62.8 (10.3)	61.8 (9.3)
Sex^a^			
Male	28 (59.6)	30 (69.8)	58 (64.4)
Female	19 (40.4)	13 (30.2)	32 (35.6)
Ethnicity^a^			
Asian	5 (10.6)	0 (0)	5 (5.6)
Black	23 (48.9)	19 (44.2)	42 (46.7)
White	18 (38.3)	23 (53.5)	41 (45.6)
Mixed or Other	1 (2.1)	1 (2.3)	2 (2.1)
Baseline measurements, mean (SD)			
Weight (kg)	101.1 (19.2)	103.0 (20.6)	102.0 (19.8)
Waist circumference (cm)	118.3 (12.7)	119.7 (13.1)	118.9 (12.8)
Seated brachial systolic blood pressure (mmHg)	133.8 (19.8)	131.6 (14.9)	132.7 (17.6)
Seated brachial diastolic blood pressure (mmHg)	78.6 (9.1)	75.2 (8.0)	77.0 (8.7)
Seated brachial pulse rate (bpm)	75.6 (12.4)	79.9 (13.5)	77.7 (13.1)
Fasting glucose (mmol/L)	10.7 (5.2)	10.2 (3.9)	10.5 (4.6)
Serum creatinine (umol/L)	106.8 (39.1)	98.1 (36.2)	102.6 (37.8)
Total cholesterol (mmol/L)	4.4 (1.3)	3.8 (0.9)	4.1 (1.2)
Triglycerides (mmol/L)	1.7 (1.0)	1.7 (1.0)	1.7 (1.0)
HDL cholesterol (mmol/L)	1.3 (0.4)	1.2 (0.3)	1.2 (0.4)
LDL cholesterol (mmol/L)	2.4 (1.1)	1.9 (0.7)	2.1 (0.9)
HbA1c (%)	9.3 (1.7)	9.0 (1.7)	9.2 (1.7)
Ao‐PWV mean of three measures (m/s)	9.4 (3.0)	9.4 (2.3)	9.4 (2.7)
Central systolic blood pressure mean of three measures (mmHg)	120.7 (17.4)	116.6 (16.0)	118.7 (16.8)
Central diastolic blood pressure mean of three measures (mmHg)	79.8 (8.8)	76.1 (8.4)	78.0 (8.8)
Augmentation index mean of three measures	18.4 (8.8)	14.8 (11.2)	16.7 (10.1)

Abbreviations: Ao‐PWV, aortic pulse wave velocity; CKD, chronic kidney disease; eGFR, estimated glomerular filtration rate; Hb, haemoglobin; HbA1c, glycated haemoglobin A1c; SD, standard deviation.

^a^ Sex and ethnicity are reported as number (%).

At baseline, the mean (SD) body weight was 101.1 kg (19.2) in the treatment (lixisenatide) group, 103.0 kg (20.6) in the placebo group, and 102.0 kg (19.8) overall. Waist circumference was 118.3 cm (12.7), 119.7 cm (13.1), and 118.9 cm (12.8) in the treatment, placebo, and overall groups, respectively.

Baseline seated brachial SBP averaged 133.8 mmHg (19.8) in the treatment group, 131.6 mmHg (14.9) in the placebo group, and 132.7 mmHg (17.6) overall. Corresponding baseline DBP was 78.6 mmHg (9.1), 75.2 mmHg (8.0), and 77.0 mmHg (8.7). Seated brachial pulse rate was 75.6 bpm (12.4) in the treatment group, 79.9 bpm (13.5) in the placebo group, and 77.7 bpm (13.1) overall.

The mean eGFR at baseline was 62.5 mL/min/1.73 m^2^ (23.0) in the treatment group, 71.9 mL/min/1.73 m^2^ (29.2) in the placebo group, and 67.0 mL/min/1.73 m^2^ (26.4) overall. Baseline fasting glucose concentrations were 10.7 mmol/L (5.2), 10.2 mmol/L (3.9), and 10.5 mmol/L (4.6) in the treatment, placebo, and overall groups, respectively.

Total cholesterol at baseline was 4.4 mmol/L (1.3) in the treatment group, 3.8 mmol/L (0.9) in the placebo group, and 4.1 mmol/L (1.2) overall. Triglyceride levels were consistent across groups at 1.7 mmol/L (1.0). HDL cholesterol was 1.3 mmol/L (0.4) in the treatment group, 1.2 mmol/L (0.3) in the placebo group, and 1.2 mmol/L (0.4) overall, while LDL cholesterol was 2.4 mmol/L (1.1), 1.9 mmol/L (0.7), and 2.1 mmol/L (0.9), respectively.

With regard to baseline HbA1c level, it was 9.3% (1.7) in the treatment group and 9.0% (1.7) in the placebo group, with an overall mean of 9.2%. Baseline haemoglobin concentration was 131.9 g/L (13.8) in the treatment group, 132.9 g/L (16.1) in the placebo group, and 132.4 g/L (14.9) overall.

Table 1 reports baseline measures for Ao‐PWV and other vascular endpoints/arterial stiffness measures. At baseline, the mean Ao‐PWV, calculated as the average of three measurements, was similar between the two groups: 9.4 (3.0) m/s in the Lixisenatide group and 9.4 (2.3) m/s in the placebo group, with an overall mean of 9.4 (2.7) m/s across all participants.

Baseline central SBP, also averaged over three measures, was slightly higher in the lixisenatide group at 120 (17.4) mmHg, 116.6 (16.0) mmHg in placebo, with a total cohort mean of 118.7 (16.8) mmHg. There were no missing baseline data for HbA1c or other variables reported in Table 1.

At baseline, central DBP was 79.8 (8.8) mmHg in the lixisenatide group, 76.1 (8.4) mmHg in the placebo group (76.1 ± 8.4 mmHg), with an overall mean of 78.0 (8.8) mmHg.

The AIx, derived as the mean of three readings, was 18.4 (8.8) mmHg in the lixisenatide group and 14.8 (11.2) in placebo, with a pooled mean of 16.7 (10.1) mmHg across the study population.

Over the 24‐week duration of the study Ao‐PWV did not differ significantly between groups. Ao‐PWV (mean ± SD) did not change significantly from baseline after 24 weeks of treatment with a mean (95% confidence intervals) 9.65 m/s (9.17, 10.13) with lixisenatide and 9.96 m/s (9.45, 10.46) with placebo, *p* = 0.378.

We did not observe any significant changes with treatment versus placebo in a panel of cardio‐renal biomarkers including sKlotho, albuminuria, central SBP, central DBP, seated brachial SBP, seated brachial DBP, weight and eGFR (Table 2). HbA1c fell significantly only with lixisenatide (Table 2) as expected. We also did not observe any significant changes in either group on several clinical and biochemical measures and markers (Table 2).

**TABLE 2:** End of study values in primary (aortic pulse wave velocity) and selected secondary endpoints in people with T2D and CKD randomised to lixisenatide and placebo.

Endpoint	Lixisenatide (95% CI)	Placebo (95% CI)	*p*‐Value (95%CI)
Aortic pulse wave velocity (m/s) (Ao‐PWV)	9.65 (9.17, 10.13)	9.96 (9.45, 10.46)	0.38 (−0.39, 1.01)
Albuminuria (AER, mcg/min)	449.9 (253.5, 646.3)	208.3 (6.9, 409.6)	0.09 (−41.2, 524.6)
Central systolic BP (mmHg)	121.9 (117.9, 125.9)	118.1 (113.9, 122.3)	0.19 (−1.9, 9.6)
Central diastolic BP (mmHg)	78.8 (76.8, 80.8)	78.1 (75.9, 80.2)	0.60 (−2.1, 3.7)
Brachial systolic BP (mmHg)	136.6 (132.3, 140.9)	133.5 (128.9, 137.9)	0.32 (−3.1, 9.3)
Brachial diastolic BP (mmHg)	78.5 (76.5, 80.4)	77.4 (75.4, 79.5)	0.48 (−1.9, 3.9)
Weight (kg)	100.6 (98.6, 102.5)	100.6 (98.5, 102.6)	0.99 (−3.1, 3.1)
eGFR (mL/min/1.73 m^2^)	67.8 (65.4, 70.3)	66.1 (63.6, 68.7)	0.34 (−1.8, 5.3)
HbA1c (%)	8.9 (8.6, 9.3)	9.6 (9.1, 9.9)	0.04 (−1.32, −0.03)

*Note*: *P* value (95% CI between treatment groups). Values are least‐squares means LSM (95% CI) from ANCOVA adjusting for baseline values.

Abbreviations: ANCOVA, analysis of covariance; CIs, confidence intervals; CKD, chronic kidney disease; eGFR, estimated glomerular filtration rate; Hb, haemoglobin; HbA1c, glycated haemoglobin A1c; SD, standard deviation.

HbA1c and AER were log transformed for anlaysis; values shown are back‐transformed geometric means.

There were no deaths during the study and overall, lixisenatide was well tolerated with no serious adverse events related to the drug reported (see Tables S1, S2).

## 4 DISCUSSION

In people with CKD and T2D, a 24‐week treatment with the shorter acting GLP‐1 RA lixisenatide had no significant effect on Ao‐PWV, central arterial blood pressure, albuminuria or other markers of arterial ageing. As expected, a significant reduction in HbA1c was noted only with lixisenatide as compared to placebo. Certain longer acting GLP‐1 RA reduce CVD risk with multiple mechanisms and pathways being proposed for these effects [26, 27] with Ao‐PWV being one such mechanism. In a recent network meta‐analysis on the effect of glucose lowering agents on arterial stiffness (GLP‐1 RA dataset mainly consisted of liraglutide and dulaglutide) it was shown that as a class, GLP‐1 RA were associated with lower Ao‐PWV versus placebo [21, 22]. The lack of improvement in Ao‐PWV in our study may be explained by lack of power or the actual absence of such a biological effect of lixisenatide on Ao‐PWV.

There are limited and conflicting data on the impact of GLP‐1 RA on arterial ageing and stiffness. Randomised controlled trials on GLP‐1 RA and Ao‐PWV have produced contradictory data, with some studies using longer acting agents such as semaglutide, dulaglutide and liraglutide demonstrating a beneficial effect, while others did not [28, 29, 30, 31]. A recent systematic review confirmed this inconsistency of data within the GLP‐1 RA class on Ao‐PWV [32]. The studies included in this review were between 12 and 52 weeks duration and the cohorts consisted of people with T2D, who were predominantly male and where ethnicity was reported, predominantly white Caucasian [32]. Two other meta‐analyses demonstrated a positive impact of GLP‐1 RA on Ao‐PWV. Batzias et al. included randomised controlled trials (RCTs) that showed reduction in Ao‐PWV of 1.5 to 1.9 m/s in substantially smaller populations than in our study (sample sizes *n* = 30 to 32) [21]. Kim et al. reported a pooled mean reduction of 0.5 m/s and also included RCTs of smaller sample sizes (*n* = 22 to 56) [22].

The evaluation of lixisenatide in acute coronary syndrome (ELIXA) CVOT in people with T2D diabetes and coronary artery disease, demonstrated that lixisenatide did not have any significant impact on CV endpoints [33]. With this in mind, it is perhaps unsurprising that lixisenatide did not show improvements in Ao‐PWV, however, it is important to also acknowledge that lack of improvement in Ao‐PWV may not necessarily explain neutral CV outcomes in this trial which contrasts with other GLP‐1 RA with beneficial CV outcomes.

Contrary to our results with lixisenatide, there is evidence for reductions in Ao‐PWV with other GLP‐1 RA, especially those which are longer acting and with demonstrated cardio‐renal benefits [2, 21, 28, 34, 35]. However, we do acknowledge that there is inconsistency in the data with GLP‐1 RA (of varying duration and formulation) with some meta analyses and studies reporting a reduction in Ao‐PWV while others showed no significant effect on Ao‐PWV or similar indices of arterial stiffness [21, 22, 30, 36]. Differences in study population, duration of treatment and baseline cardio‐renal risk may explain some or most of this observed discordance in the literature.

Several studies using longer acting agents such as semaglutide, dulaglutide and liraglutide demonstrated a beneficial effect on markers of arterial stiffness. Semaglutide demonstrated reduction in Ao‐PWV and other markers of arterial stiffness in a study involving people with T2D and fatty liver disease (*n* = 75 in total) [37], while no effect was observed in another trial in a T2D population [36]. CVOT in people with T2D and people with obesity without T2D demonstrated reduced risk of CVD events and CVD mortality in those treated with semaglutide as compared to placebo [8, 38]. Similarly, dulaglutide has demonstrated a beneficial effect on Ao‐PWV in people with T2D in a RCT with *n* = 56 participants in each group, [29] and a positive impact on CVD endpoints in CVOT [9]. Liraglutide was also shown to reduce Ao‐PWV (−1.5 m/s) in people with T2D in some studies (*n* = 60 in total) [39] while in others it did not [30]. CVOT with liraglutide have also shown a protective effect for CVD endpoints [7]. In contrast to these GLP‐1 RA, the CVOT for lixisenatide (ELIXA) did not show any significant impact on the rate of CVD events [33]. Interestingly, the CVOT for Albiglutide (HARMONY) had a population with similar baseline characteristics, including CVD risk, to that in ELIXA [40]. Albiglutide demonstrated a positive effect on CVD outcomes whereas with lixisenatide such effect was not observed [33, 40].

There are no studies as far as we are aware reporting the role of any of GLP‐1 RA on the cardio‐renal protective anti‐ageing hormone sKlotho. sKlotho is an emerging biomarker of arterial ageing and cardio‐renal risk that plays a key role in the arterial ageing process by modulating arterial wall inflammation and calcification. sKlotho, which is expressed predominantly in the kidney, promotes phosphaturia and is a co‐factor for Fibroblast Growth Factor‐23, which is a mediator of phosphate balance and also an independent CVD risk factor in CKD in diabetes. Klotho levels in the circulation are reduced in people with CKD and diabetes, and lower levels are an independent predictor of progression of CKD [41, 42]. In our study, 24‐week treatment with lixisenatide did not have an impact on sKlotho. We also did not observe any significant impact on a panel of other biomarkers implicated in cardio‐renal disease. Of note, changes in sKlotho have been observed with RAAS inhibitors as well as SGLT‐2 inhibitors in some studies but not all [24, 43, 44]. As expected, changes in HbA1c were observed with a significant reduction noted in participants on lixisenatide treatment as compared to placebo.

There are several strengths and limitations to our study. Our original study plan and design was powered to detect at least a 1 m/s difference in Ao‐PWV with at least 104 participants in total (52 in each group) [16, 19, 20, 45, 46, 47, 48]. Recent data demonstrated that GLP‐1 RA such as dulaglutide, liraglutide and semaglutide are associated with a reduction in Ao‐PWV between 0.5 and 1.5 m/s [21, 22, 29, 35, 37]. The existing literature for GLP‐1 RA, suggested similar or indeed greater effect size to ours, even with liraglutide which is relatively shorter acting than weekly GLP‐1 RAs [39]. These studies had sample sizes similar or smaller than ours (*n* = 22 to 75). However, we acknowledge lixisenatide is significantly shorter acting than these other GLP‐1RA, with a substantially different pharmacodynamic profile and our target effect size of 1 m/s in Ao‐PWV may have resulted in reduced power. We also could not achieve the goal of 104 participants and only reached 101 of whom 90 were eligible for the final analyses due to impact of COVID‐19 pandemic which resulted in suspension and subsequent cessation of new participant recruitment. This is an important limitation which reduced the power of our study and increases the risk of type II error, although it is important to note that studies with other GLP‐1 RA had smaller sample sizes and achieved similar or larger Ao‐PWV effect sizes (up to 1.9 m/s) as discussed earlier [21, 22].

One of our strengths are that we included only people with CKD related to T2D, had a more ethnically diverse cohort as comapred to similar trials and excluded those with other causes of CKD. Furthermore, our study is one of the largest and longest RCTs conducted to date evaluating the effect of GLP‐1 RA on the primary endpoint of arterial stiffness. The limitations of our study are that this was a single centre study in an urban cohort of people with CKD and T2D and our results may not be applicable to all cohorts of people with diabetes. As this was a proof‐of‐concept trial our study may be underpowered to detect the changes we estimated which were extrapolated from other studies. Another limitation of our study was that only 35.6% of the study cohort were women. As described previously we did not observe any impact of lixisenatide on several exploratory secondary endpoints, such as central arterial pressure, AIx and albumin excretion that are biomarkers and clinical predictors of enhanced cardio‐renal risk. The study was, however, not designed or powered to detect changes in these multiple secondary endpoints. As this was a RCT baseline comparisons would between the two groups are not recommended and may be not particularly meaningful [49], however, potential differences in baseline variables are not excluded and could have affected the outcomes observed. Finally, the low background use of SGLT‐2 inhibitors (<15%) and lack of use of nsMRA  may limit generalisability of the study. We also acknowledge that use of SGLT‐2 inhibitors and nsMRA in recent larger clinical trials is also often low; for example, SGLT‐2 inhibitor use was 15.6% in FLOW trial [13] and ~31% in the recent SURPASS CVOT (MRA use <10% in SURPASS CVOT) [50]. Similar or even lower use is seen in real world for both SGLT‐2 inhibitors and nsMRA despite guideline recommendations [51, 52, 53].

Missing data were handled using LOCF, which represents a conservative approach in the context of this trial. Importantly, no COVID‐19‐related missing follow‐up occurred in this trial, and the proportion of post‐baseline missing data was small, reducing the potential for bias due to LOCF compared with settings with extensive dropout. LOCF has been used in diabetes related clinical trials with limited missingness when outcomes change relatively slowly over time [54, 55]. Although LOCF has known limitations and strong assumptions, chiefly that an individual's outcome would remain unchanged after dropout, it remains a frequently used approach in clinical research when missing data are minimal and not driven by external factors [56].

The 24‐week treatment period with lixisenatide is longer than many other similar, proof of concept trials and indeed changes in Ao‐PWV have been observed with other interventions of similar or shorter duration (12 to 26 weeks) [21, 34, 35]. Larger trials are needed to definitively exclude the impact of GLP‐1 RA on Ao‐PWV.

In conclusion, in our study of people with T2D and CKD, 24‐week treatment with the shorter acting GLP‐1 RA lixisenatide had no significant effect on Ao‐PWV, central arterial blood pressure or other biomarkers of CV risk. The lack of improvement observed may not necessarily fully explain neutral CV outcomes observed with lixisenatide, which contrasts with other GLP‐1 RA with beneficial CV outcomes. It is difficult to disentangle whether the lack of improvement in Ao‐PWV is due to lack of power in our study to detect smaller, yet potentially meaningful changes, or true absence of an effect. However, studies using other longer acting GLP‐1 RA of similar duration (−24 weeks) and smaller sample sizes have shown a positive impact on Ao‐PWV. Further studies are needed to further understand the potential mechanisms that may explain discordance in CVD outcomes observed with the GLP‐1 RA class and the related clinical relevance.

## AUTHOR CONTRIBUTIONS

Janaka Karalliedde, Luigi Gnudi designed the research study, interpreted the data and drafted the article. Panagiotis Pavlou, Nikolaos Fountoulakis, Dimitra Stathi, Maria Flaquer, Antonella Corcillo collected and interpreted the data and contributed to the manuscript. Aicha Goubar, Salma Ayis contributed and led on data analysis and interpretation. All authors have reviewed the article and approved the final draft.

## CONFLICT OF INTEREST STATEMENT

The authors declare that there is no duality/conflict of interest associated with the manuscript.

## Supporting information


**Table S1:** Adverse events.
**Table S2:** Serious adverse events.
**Figure S1:** CONSORT diagram.

## Data Availability

The dataset generated and/or analyzed during the study is stored in a non‐publicly available repository (MACRO system).
