# Supplementary material for: Effect of lixisenatide on arterial stiffness in people with type 2 diabetes and kidney disease: Results of a randomised controlled trial
Source: Diabetes Obes Metab. 2026 Feb 18;28(4):2953–61. doi: 10.1111/dom.70481 (PMC12992176; doi:10.1111/dom.70481)
Supplement: Supplementary file 1 — Table S1: Adverse events. Table S2: Serious adverse events. Figure S1: CONSORT diagram. [file DOM-28-2953-s001.docx]

## Supplementary Material:

## Supplement Table 1. Adverse Events.

| Event | Lixisenatide N (%) | Placebo N (%) | Total N (%) |
| --- | --- | --- | --- |
| Participants with ≥1 adverse event | 35 (68.6%) | 32 (64.0%) | 67 (66.3%) |
| Adverse events leading to discontinuation | 3 (5.9%) | 2 (4.0%) | 5 (5.0%) |
| Nausea | 6 (11.8%) | 5 (10.0%) | 11 (10.9%) |
| Vomiting | 3 (5.9%) | 2 (4.0%) | 5 (5.0%) |
| Diarrhea | 4 (7.8%) | 3 (6.0%) | 7 (6.9%) |
| Abdominal pain | 2 (3.9%) | 2 (4.0%) | 4 (4.0%) |
| Dyspepsia | 1 (2.0%) | 1 (2.0%) | 2 (2.0%) |
| Decreased appetite | 2 (3.9%) | 1 (2.0%) | 3 (3.0%) |
| Constipation | 2 (3.9%) | 1 (2.0%) | 3 (3.0%) |
| Musculoskeletal pain | 3 (5.9%) | 2 (4.0%) | 5 (5.0%) |
| Infections (respiratory, urinary) | 4 (7.8%) | 5 (10.0%) | 9 (8.9%) |
| Cardiovascular AE (hypertension, ACS, stroke/TIA) | 3 (5.9%) | 2 (4.0%) | 5 (5.0%) |
| Other events | 2 (3.9%) | 2 (4.0%) | 4 (4.0%) |

*Participants may be counted in more than one category. Percentages are based on total N per arm (Lixisenatide=51, Placebo=50).

*Abbreviations: ACS=Acute Coronary Syndrome, TIA=Transient Ischemic Attack, AE=Adverse Events*

## Supplement Table 2. Serious Adverse Events.

| Event | Lixisenatide N (%) | Placebo N (%) | Total N (%) |
| --- | --- | --- | --- |
| Participants with ≥1 serious adverse event | 5 (9.8%) | 3 (6.0%) | 8 (7.9%) |
| Cardiovascular SAEs (ACS, MI, stroke/TIA) | 2 (3.9%) | 2 (4.0%) | 4 (4.0%) |
| Infections | 1 (2.0%) | 1 (2.0%) | 2 (2.0%) |
| Deaths | 0 (0.0%) | 0 (0.0%) | 0 (0.0%) |
| Discontinuations due to SAE | 1 (2.0%) | 1 (2.0%) | 2 (2.0%) |

*Participants may be counted in more than one category. Percentages are based on total N per arm (Lixisenatide=51, Placebo=50).

*Abbreviations: ACS=Acute Coronary Syndrome, TIA=Transient Ischemic Attack, MI=Myocardial Infarction, SAE=Serious Adverse Events*

## Supplementary Figure 1: CONSORT diagram

Assessed for eligibility (n=143)

**Enrolment**

Allocated to

Lixisenatide (n= 51)

Excluded (n=42)

- Failure to meet criteria (n=28)
- Trial declined (n=5)
- Other reasons (n=9)

Randomised (n=**101)**

**Allocation**

Allocated to

Placebo (n= 50)

N=7 dropped out (preference/inability to adhere to schedule)

N= 10 dropped out (n=9 due to preference/inability to adhere to schedule, n=1 due to health reasons)

- Population analysed (n=47)
  Excluded from analysis (no post randomisation data; n=4)
- Population analysed (n=43)
  Excluded from analysis (no post randomisation data; n=7)

**Analysis**
